# Improving Braden Scale Risk Assessment Documentation: A Two-Cycle Clinical Audit at Al-Managil Teaching Hospital, Sudan

**DOI:** 10.7759/cureus.100493

**Published:** 2025-12-31

**Authors:** Hala Omer Mohammed Taha, Amani Abdallah Alhajj Abdallah, Salih Ahmed, Mohammed Abdalazeem Alsheikh Ahmed, Muram Mustafa Mohamed Mohamed, Husam Eldin Abuelgassim Hassan Balila, Sanaa Mohammedelbager Mahmoud Mursy, Mohamed Ziada, Mohamed Mahde, Malaz Siddeg Hamed Younis, Hago Ahmed Mohamedali Ahmed, Alya Elmaimona Hashim Awad Abdelmageed, Mazin Abdelrahman Elhag, Tasneem Mohamed Yousif Mohamed, Yusuf M. Y. Idris, Amr Elbanna Mohammed Aly, Mohamed Elsayed Abdelhalim Hassan, Adam Salama Mohamed Adam, Elwathig Abdalla, Montaser Mohammed Ahmed Tambl Ably

**Affiliations:** 1 Internal Medicine, Dongola Specialized Hospital, Dongola, SDN; 2 General Surgery, Al-Managil Teaching Hospital, Al-Managil, SDN; 3 Medicine, Omdurman Islamic University, Omdurman, SDN; 4 General Surgery, Dongola Teaching Hospital, Dongola, SDN; 5 General Surgery, El-Gadarif Teaching Hospital, El-Gadarif, SDN; 6 Medicine, Nile College, Khartoum, SDN; 7 Public Health, International University of Africa, Khartoum, SDN; 8 General and Vascular Surgery, United Lincolnshire Teaching Hospitals NHS Trust (Pilgrim Hospital), Boston, GBR; 9 Plastic Surgery, Tajmeel Medical Centre, Sharjah, ARE; 10 Neurological Surgery, Ribat Teaching Hospital, Khartoum, SDN; 11 Orthopaedics and Traumatology, Klinikum Dahme Spreewald GmbH, Luebben, DEU; 12 Surgery, King Saud Medical City, Riyadh, SAU; 13 Emergency Medicine, Al-Managil Teaching Hospital, Al-Managil, SDN; 14 Emergency Medicine, Military Hospital, Khartoum, SDN; 15 General and Colorectal Surgery, South Tipperary General Hospital (STGH), Clonmel, IRL; 16 Surgery, Almanagil Teaching Hospital, Al-Managil, SDN; 17 Orthopaedic Surgery, Jalan Bani Buali Hospital, OMN; 18 Orthopaedics, Sudan Medical Specialization Board, Khartoum, SDN

**Keywords:** braden scale, clinical audit, documentation, nursing practice, pressure injury, quality improvement, risk assessment

## Abstract

Background: Accurate Braden Scale documentation is essential for early identification of patients at risk for pressure injuries. Initial observations at Al-Managil Teaching Hospital in Al Managil, Sudan, indicated poor compliance with the Braden Scale assessment and documentation.

Objectives: To evaluate the completeness of Braden Scale documentation before and after a targeted educational and documentation-standardization intervention.

Methods: A closed-loop clinical audit was conducted in two cycles, each reviewing 51 patient records. Cycle 1 (retrospective) was performed on 16 May 2025. A two-month intervention consisting of staff education and implementation of a standardized Braden documentation form was introduced, followed by a prospective three-month Cycle 2. Data were analyzed using descriptive statistics and chi-square testing for documentation improvement.

Results: Baseline documentation was markedly incomplete, with 0% recording of all Braden subscales, total score, and risk level. After the intervention, Cycle 2 achieved 100% completion across all Braden components and significant improvements in patient identifiers and administrative fields (p < 0.001 for most parameters). The largest improvements were observed in subscale documentation, risk scoring, and action notes.

Conclusion: Targeted education combined with a standardized Braden Scale tool resulted in substantial and statistically significant improvements in documentation quality. These low-cost interventions can strengthen pressure-injury prevention practices in resource-limited settings. Continued monitoring and re-auditing are recommended to sustain compliance and assess clinical impact.

## Introduction

Pressure injuries remain a significant cause of preventable morbidity and mortality in hospitalized patients, particularly in settings where early detection and preventive measures are inconsistently applied. Their development is associated with prolonged hospitalization, increased healthcare expenditure, and considerable patient suffering. A recent global systematic review involving more than 2.5 million hospitalized adults reported a pooled pressure injury prevalence of 12.8% and highlighted substantial variation across regions, underscoring the persistent burden of these largely preventable conditions despite improvements in clinical care [1].

To address this burden, international expert groups have established comprehensive, evidence-based guidelines emphasizing structured risk assessment and regular skin inspection as core components of pressure injury prevention. According to the 2019 International Guideline issued by the European Pressure Ulcer Advisory Panel (EPUAP), the National Pressure Injury Advisory Panel (NPIAP), and the Pan Pacific Pressure Injury Alliance (PPPIA), standardized assessment using validated tools should be performed for all at-risk patients and integrated into routine documentation and care planning [2]. These guidelines emphasize that effective prevention relies on both accurate assessment and consistent documentation.

Among the validated assessment tools, the Braden Scale is one of the most widely adopted, incorporating six domains, namely, sensory perception, moisture, activity, mobility, nutrition, and friction/shear, to generate a risk score that guides targeted preventive interventions. Its foundational validation study demonstrated strong inter-rater reliability and good predictive validity across care settings, supporting its widespread clinical use as a structured, standardized approach to early risk detection [3].

Importantly, the usefulness of the Braden Scale in clinical practice is closely tied to how accurately and consistently it is applied. Recent research comparing Braden subscale scores with objective, sensor-based movement measurements in long-term care residents showed that accurately completed assessments reflect real patient mobility patterns and provide clinically meaningful insight for personalizing prevention strategies [4]. Conversely, incomplete or inaccurate documentation limits early recognition of risk, weakens communication across care teams, and contributes to preventable pressure injuries.

Clinical audit plays a central role in evaluating how well evidence-based practices, such as the Braden Scale assessment and documentation, are implemented within healthcare settings. Quality improvement initiatives using structured audits have demonstrated substantial reductions in hospital-acquired pressure injuries when preventive bundles, education, and consistent documentation are integrated into routine care. For instance, a multidisciplinary program in a tertiary cardiac hospital in Qatar achieved an 83.5% reduction in hospital-acquired pressure injuries over four years through structured prevention strategies and continuous monitoring [5]. This highlights the value of local audits, particularly in resource-limited settings, to assess current practice, identify gaps, and guide targeted interventions aimed at improving pressure injury prevention and documentation quality. Accordingly, the objective of this study was to evaluate the completeness of Braden Scale documentation among pediatric inpatients before and after a targeted educational and documentation-standardization intervention, with specific attention to subscale recording, total scores, and assigned risk levels.

## Materials and methods

Study design

A retrospective, closed-loop clinical audit was conducted over a six-month period at Al-Managil Teaching Hospital in Al-Managil, Sudan, to evaluate and improve the completeness of Braden Scale documentation among pediatric inpatients. The audit followed the standard audit cycle, beginning with a baseline assessment (Cycle 1), followed by a targeted educational intervention, and concluding with a prospective reassessment (Cycle 2).

Study setting

The audit was performed in the pediatric ward of Al-Managil Teaching Hospital, a regional referral center where the Braden Scale is the standard tool for assessing pressure injury risk. Nursing staff are expected to complete a full Braden assessment for all admitted pediatric patients at the time of admission. The audit sought to determine the extent to which routine clinical documentation adhered to this requirement.

Audit timeline

Cycle 1 was conducted retrospectively on 16 May 2025 and evaluated documentation practices prior to any intervention. This was followed by a two-month intervention phase extending from mid-May to mid-July 2025, during which staff education and documentation-focused improvements were implemented. Cycle 2 was subsequently performed prospectively over a three-month period (mid-July to mid-October 2025), applying the same criteria and data-collection procedures to reassess documentation quality and measure the effect of the intervention.

Study population and sample size

The audit included 51 pediatric inpatient records in each cycle. All medical records in which the Braden Scale assessment should have been completed at admission were eligible for inclusion. No exclusions were applied in order to capture an accurate representation of routine documentation practices within the ward.

Audit standards and evaluation criteria

Audit standards were based on international pressure injury prevention guidelines and institutional requirements for Braden Scale documentation. Each medical record was evaluated for the completeness of all required components. These included patient identifiers (name, age, hospital number, and bed number), admission information (ward or unit, consultant name, and admission date), and the clinical diagnosis. The audit also examined whether all six Braden Scale subcomponents-sensory perception, moisture, activity, mobility, nutrition, and friction or shear-were documented. Additionally, the total Braden score, assigned risk category, documentation of actions taken or preventive measures, and the assessor’s name or initials were reviewed. A documentation field was considered “completed” only when all required information within that category was fully and accurately recorded.

Data collection procedure

For Cycle 1, retrospective data were extracted from physical patient records dated prior to 16 May 2025. A structured checklist was used to ensure uniformity during data extraction, and findings were recorded using a purpose-built Google Forms tool (Google Inc., Mountain View, CA). Cycle 2 data were collected prospectively over the subsequent three months using the same checklist and documentation standards to ensure direct comparability between cycles. Google Forms automatically generated a digital spreadsheet containing all entries, which was used for analysis.

Intervention between audit cycles

A targeted two-month quality-improvement intervention was implemented after Cycle 1. This intervention consisted of structured educational sessions for pediatric and nursing staff on correct Braden Scale scoring, interpretation, and documentation; reinforcement of documentation expectations during shift handovers and ward rounds; clarification of common documentation errors identified in Cycle 1; and emphasis on linking Braden scores to appropriate preventive measures. The hospital’s Braden documentation form was standardized to ensure greater clarity and ease of use. The aim of the intervention was to enhance staff awareness, accuracy, and consistency in completing Braden assessments.

Data analysis

Data extracted from Google Forms were exported into a spreadsheet for statistical analysis. Each documentation item was coded dichotomously as either “completed” or “not completed.” Completion rates were expressed as percentages for each cycle. Improvements between Cycle 1 and Cycle 2 were calculated by comparing these percentages.

To determine whether the observed differences in documentation completeness were statistically significant, chi-square (χ²) tests were applied to compare proportions between cycles for each documentation parameter. The level of statistical significance was set at p < 0.05. All p-values, χ² values, and corresponding improvements were reported for individual documentation elements, enabling objective assessment of the intervention’s impact.

Ethical considerations

This audit was conducted in accordance with institutional ethical requirements and clinical governance standards of Al-Managil Teaching Hospital under reference number MTH-1795. The audit involved retrospective and prospective review of routine clinical documentation only; no direct patient contact occurred, and all data were fully anonymized to ensure confidentiality.

## Results

A total of 51 pediatric records were reviewed in each cycle. The baseline audit revealed substantial deficiencies in Braden Scale documentation, particularly in core assessment elements such as subscale scoring, total score calculation, risk categorization, and notes on preventive actions. Following the intervention, Cycle 2 demonstrated marked improvement across all documentation parameters.

General patient identifiers such as name, age, location, and consultant information showed dramatic increases, with documentation completeness rising from less than one-third in Cycle 1 to more than 96% in Cycle 2. Administrative identifiers, most notably the hospital number and bed number, also improved significantly, although the bed number field showed the smallest overall gain.

Clinical information displayed similar improvement. Documentation of diagnosis increased from 76.5% to 100%, while the date field rose sharply from 3.9% to 96.1%. The most notable improvements were seen in the Braden Scale components themselves. All six subscales, the total score, the assigned risk level, assessor identification, and documentation of actions taken increased from 0% in Cycle 1 to full completion (100%) in Cycle 2.

Statistical testing confirmed that these improvements were highly significant. With the exception of the bed number parameter, all other variables demonstrated extremely large chi-square values accompanied by p-values < 0.0001, indicating strong evidence of an intervention effect. The Braden subscales and related fields reached the highest possible significance, reflecting the transition from complete absence of documentation to universal completion.

Overall, the re-audit demonstrated a comprehensive, organization-wide improvement in Braden Scale assessment and documentation practices, suggesting that the targeted educational intervention and heightened awareness successfully addressed the gaps identified in the initial cycle (Table 1).

**Table 1:** Comparison of Braden Scale Documentation Between Cycle 1 and Cycle 2 (n = 51 per cycle) Comparison of Braden Scale documentation completeness between the first and second audit cycles. Each parameter reflects whether the corresponding item was fully recorded in the patient file. Cycle 1 was conducted retrospectively on 16 May 2025, followed by a two-month educational intervention, and Cycle 2 was collected prospectively over three months. Percentages represent the proportion of records in which the parameter was documented. Statistical significance was assessed using chi-square (χ²) tests comparing completed versus not-completed documentation for each parameter. A p-value < 0.05 was considered statistically significant.

Parameter	Cycle 1 (n=51)	Cycle 2 (n=51)	χ² (df = 1)	p-value	Effect size (Cramer’s V)
Patient name	14 (27.5%)	50 (98.0%)	51.38	7.62×10⁻¹³	0.71 (large)
Age	10 (19.6%)	50 (98.0%)	61.56	4.29×10⁻¹⁵	0.78 (large)
Ward/unit	5 (9.8%)	49 (96.1%)	72.76	1.46×10⁻¹⁷	0.84 (large)
Consultant	6 (11.8%)	49 (96.1%)	69.6	7.25×10⁻¹⁷	0.82 (large)
Hospital No.	0 (0.0%)	35 (68.6%)	50.28	1.33×10⁻¹²	0.70 (large)
Bed No.	0 (0.0%)	9 (17.6%)	7.8	0.00523	0.28 (small–medium)
Diagnosis	39 (76.5%)	51 (100.0%)	11.43	0.00072	0.33 (medium)
Date	2 (3.9%)	49 (96.1%)	82.98	8.29×10⁻²⁰	0.90 (large)
Sensory score	0 (0.0%)	51 (100.0%)	98.04	4.10×10⁻²³	1.00 (perfect)
Moisture	0 (0.0%)	51 (100.0%)	98.04	4.10×10⁻²³	1.00 (perfect)
Activity	0 (0.0%)	51 (100.0%)	98.04	4.10×10⁻²³	1.00 (perfect)
Mobility	0 (0.0%)	51 (100.0%)	98.04	4.10×10⁻²³	1.00 (perfect)
Nutrition	0 (0.0%)	51 (100.0%)	98.04	4.10×10⁻²³	1.00 (perfect)
Friction/shear	0 (0.0%)	51 (100.0%)	98.04	4.10×10⁻²³	1.00 (perfect)
Total score	0 (0.0%)	51 (100.0%)	98.04	4.10×10⁻²³	1.00 (perfect)
Risk level	0 (0.0%)	51 (100.0%)	98.04	4.10×10⁻²³	1.00 (perfect)
Actions taken/Notes	0 (0.0%)	51 (100.0%)	98.04	4.10×10⁻²³	1.00 (perfect)
Assessor	0 (0.0%)	51 (100.0%)	98.04	4.10×10⁻²³	1.00 (perfect)

## Discussion

This closed-loop clinical audit demonstrated that a structured intervention, combining clinician education with a standardized Braden Scale documentation form, resulted in substantial and statistically significant improvements in the completeness of pressure-injury risk documentation. During the first cycle, core patient identifiers and administrative data were frequently missing, and none of the Braden subscales, total scores, or preventive actions were documented. This pattern of incomplete and inaccurate documentation is consistent with the findings of Gunningberg and Ehrenberg, who reported significant discrepancies between pressure-ulcer documentation and direct patient examination, with nursing records frequently under-reporting risk and preventive measures [6].

Following the intervention, documentation completeness improved markedly across nearly all items, especially demographic identifiers and clinical fields such as date and diagnosis. The positive impact of educational interventions on pressure-injury prevention and documentation practices is well supported in the literature. In Gunningberg’s evaluation of a Swedish nurse-training program, staff education resulted in improved preventive routines and better recording of pressure ulcer-related information [7]. Our findings align with this evidence, demonstrating that targeted education and a simplified documentation format can rapidly improve compliance, even in resource-constrained clinical settings.

One of the most striking improvements in our audit was the complete transition from 0% Braden documentation in Cycle 1 to 100% completion of all six subscales, total score, and risk level in Cycle 2. Accurate and complete Braden scoring is of high clinical value. A systematic review and meta-analysis by Huang et al. reported strong predictive validity of the Braden Scale, with clinically meaningful accuracy for identifying at-risk adult patients [8]. Additionally, modern validation work by Kennerly et al. demonstrated that Braden subscale scores, particularly activity and mobility, correlate with objective sensor-based patient-movement data, reinforcing the clinical usefulness of detailed subscale scoring rather than superficial or incomplete documentation [4]. Thus, the improvements observed in our audit reflect not only better documentation standards but also enhanced alignment with evidence-based risk assessment.

Broader quality-improvement literature also supports the concept that structured documentation and multidisciplinary prevention bundles can reduce pressure-injury incidence. For example, a hospital-wide quality improvement program in Qatar achieved an over 80% reduction in pressure injuries through systematic staff training, standardized risk assessment, and continuous monitoring [5]. These findings emphasize the importance of embedding consistent assessment tools within routine workflows, supported by feedback loops and leadership engagement.

Despite the substantial gains, some documentation challenges persist. particularly regarding administrative fields such as bed number and hospital number. This is not unique to our setting: a recent retrospective review of medical records in a Swedish internal-medicine ward found that pressure-ulcer risk assessments and ulcer documentation were extremely low (only 2.1% in care plans and 4.7% in nursing notes), and many care events lacked defined ulcer status or risk assessment, suggesting that documentation deficiencies remain pervasive in hospital records globally [9,10].

Moreover, the literature suggests that audit-and-feedback approaches combining regular chart reviews, staff feedback, and preventive-care audits can produce sustained improvements over time. In a multicenter longitudinal study across nursing homes over two years, such a program significantly improved adherence to repositioning and use of anti-decubitus surfaces, with a corresponding reduction in pressure ulcer prevalence [11].

Implementation and quality-improvement studies further highlight that the effectiveness of pressure-injury prevention depends on multifaceted strategies: staff education, structured documentation templates, multidisciplinary involvement, and consistent monitoring are more successful than isolated interventions [12,13].

In addition to preventing physical harm, robust documentation has broader implications: accurate and comprehensive nursing records have been shown to correlate significantly with continuity of care, interprofessional communication, and medico-legal accountability, reinforcing the value of documentation quality beyond mere risk assessment [14].

Finally, documentation quality is closely tied to interprofessional communication, medico-legal protection, and continuity of care. A systematic review by Bunting and de Klerk found that multifaceted strategies, including education, structured forms, feedback, and audit, are most effective in improving compliance with documentation guidelines in hospital settings [15]. This supports the approach used in our audit, wherein structured forms and targeted teaching produced rapid improvements. To sustain these gains, ongoing audit cycles, refresher training, and potential integration into electronic systems may be needed.

In summary, this audit demonstrates that simple, targeted quality-improvement measures, specifically staff education and a standardized Braden documentation tool, can yield rapid and profound improvements in documentation completeness. Although limited to one institution and focused on documentation rather than patient outcomes, the findings provide strong evidence supporting the use of closed-loop audits to enhance compliance with risk-assessment standards. As with other audit-based quality-improvement studies, the absence of a control group and the possibility of a Hawthorne effect may have influenced staff behavior during the re-audit period. In addition, the short follow-up duration precludes assessment of long-term sustainability, and improvements in documentation may not necessarily translate into measurable reductions in pressure-injury incidence. Unmeasured contextual factors, such as staffing changes or variations in patient case mix, may also have contributed to the observed improvements. Future work should evaluate the impact of improved documentation on pressure-injury incidence and assess long-term sustainability.

## Conclusions

This closed-loop audit showed that targeted staff education and a standardized Braden scale form led to major improvements in documentation quality, with all Braden subscales and risk scores increasing from 0% to full completion in the second cycle. These findings demonstrate that simple, low-cost interventions can rapidly enhance pressure-injury risk-assessment practices, even in resource-limited settings. Continued monitoring and periodic re-auditing are recommended to sustain these gains and to evaluate their impact on actual pressure-injury outcomes.
